# Detection of rabies virus RNA in dog-bite wounds in a rabies-endemic area: evidence from an observational cohort study

**DOI:** 10.1016/j.ebiom.2026.106250

**Published:** 2026-04-10

**Authors:** Carmen W.E. Embregts, Naseem Salahuddin, M. Aftab Gohar, Fouzia Naseer, Shakir Hussain, Kimberley Alblas, Jolanda J.C. Voermans, Judith Guldemeester, Bas B. Oude Munnink, Javeria Aijaz, Corine H. GeurtsvanKessel

**Affiliations:** a Department of Viroscience, Erasmus Medical Centre, Rotterdam, the Netherlands; b Infectious Disease Division, Department of Medicine, The Indus Hospital, Karachi, Pakistan; c Rabies Prevention and Training Center, Indus Hospital & Health Network, Karachi, Pakistan; d Molecular Pathology Section, Clinical Laboratories, Indus Hospital & Health Network, Karachi, Pakistan

**Keywords:** Rabies virus, Surveillance, Sequencing, Wound swabs, Dog-bite injuries

## Abstract

**Background:**

Rabies causes over 59,000 human deaths each year, primarily in endemic regions where surveillance is challenging. We report a prospective observational cohort study evaluating rabies virus (RABV) RNA detection from wound swabs of individuals bitten by dogs as a tool to strengthen surveillance and assess human exposure.

**Methods:**

We enrolled 100 individuals with unwashed, less than 24 hours-old, WHO-defined category III bite wounds at the Rabies Prevention Center, Indus Hospital, Karachi, Pakistan. Swabs were collected from wounds before and after washing. Serum samples were obtained before and 14 days after initiation of the three-visit intradermal IPC post-exposure prophylaxis (PEP) regimen. Demographic and clinical data were recorded. RABV RNA was detected using genotype-1 and pan-lyssavirus PCR assays; detection of RABV neutralising antibodies was evaluated by rabies fluorescent antibody virus neutralisation test (rFAVN). Primary outcome was RABV RNA detection; secondary outcomes included bite characteristics and virus-neutralising antibody (VNA) titres.

**Findings:**

Among 100 participants (mean age 18.9 years; 82% male), bites occurred mainly on the lower limb (34%), head (21%) and arm (15%). RABV RNA was detected in 64% of swabs by genotype-1 and 44% by pan-lyssavirus PCR; from 40/64 positive samples the RABV-N gene was sequenced and used for downstream phylogenetic analysis. Wound washing slightly decreased RNA levels. At day 14, RABV neutralising antibodies were detected in 93.8% (75/80) of the individuals (mean titre 5.8 IU/mL).

**Interpretation:**

Wound swab–based RABV RNA detection is a feasible, scalable method for rabies surveillance and exposure assessment in endemic settings. Combined with serology, it informs both viral exposure and PEP efficacy, supporting broader application in high-volume clinical care.

**Funding:**

Supported by a VENI Grant (NWO-VENI 09150162010181, to CWEE) and Off Road grant (ZonMw 04510252520092, to CWEE).

**Research in context**

**Evidence before this study**

We searched PubMed and Scopus from database inception to July 2025 using the terms “rabies,” “wound swab,” “RNA detection,” “rabies virus,” and “surveillance.” No previous studies were found assessing RABV RNA detection from wound swabs in human dog-bites. While previous work has described virus detection in animal saliva and post-mortem tissue, there is a critical gap in non-invasive, scalable tools for rabies surveillance in humans, as well as for tools to assess potential exposure to RABV.

**Added value of this study**

We show that RABV RNA can be detected in a high proportion of swabs collected from unwashed wounds within 24 hours of the bite. The sampling approach is minimally invasive and well suited for resource-limited rabies-endemic areas, as well as high-volume clinical settings like clinical PEP trials. Our study demonstrates the effect of thorough wound washing on RABV RNA detection and confirms that most patients develop robust levels of neutralizing antibodies following the three-dose intradermal IPC PEP regimen.

**Implications of all the available evidence**

Our findings demonstrate that RABV RNA detection from wound swabs may serve as a complementary tool for RABV surveillance in endemic settings, particularly where animal testing and routine surveillance are limited or absent. While the high proportion of positive swabs in this study cannot be extrapolated to the general dog-bite population due to strict inclusion criteria, it highlights the potential to monitor RABV circulation via human exposures. Molecular characterization of swab-derived viral RNA could also provide insights into circulating viral strains and inform public health strategies. When implemented thoughtfully, integrating wound swab PCR into clinical workflows could enhance understanding of rabies epidemiology and support the design of future studies on PEP efficacy.

## Introduction

Rabies, an ancient and devastating viral zoonosis, remains the infectious disease with the highest known case-fatality rate. Without prompt and appropriate post-exposure prophylaxis (PEP), rabies invariably leads to death. Since the 1950s, 18 Lyssavirus species have been identified, of which seven have been reported to cause human rabies fatalities.^1^^,^^2^ Despite this diversity, 99% of human cases worldwide are attributable to infection with rabies virus (RABV), typically contracted through the bite or scratch of an infected animal, most commonly a dog.^3^

At least 59,000 human fatalities occur each year due to rabies, most of them in low- and middle-income regions in Asia and Africa, with about half of the individuals being children under the age of 15.^4^ The global burden of this neglected disease is likely to be much higher given the profound underreporting that persists in many endemic areas,^5^^,^^6^ and the fact that two-thirds of the world's population lives in rabies-endemic areas.^3^

Although rabies is endemic in Pakistan, it remains a low-priority disease. Reliable data on human and animal rabies deaths are scarce, with rough estimates ranging from 2000 to 5000 human deaths annually. The country has a large population of free-roaming, unowned dogs in both urban and rural areas, but no systematic epidemiological surveys of dog bites or rabies incidence have been conducted. Rabies is a non-notifiable disease in Pakistan and receives limited legislative or public health support. Most rabies deaths are reported via lay media, often from peri-urban or rural areas with poor access to medical care, while many individuals at risk seek treatment from faith healers.

The Indus Hospital & Health Network (IHHN) is a network of primary, secondary, and tertiary care facilities providing free-of-cost healthcare. Its first hospital in Karachi, The Indus Hospital (TIH) in Korangi town, offers free post-exposure prophylaxis, including wound washing, rabies vaccine, and rabies immunoglobulin when indicated. TIH also provides PEP training to healthcare workers from other institutions, positioning it as a critical centre for rabies prevention and management in the region.

A major obstacle to rabies control is its complex pathogenesis and the lack of accessible, reliable diagnostics and surveillance methods. Following exposure, RABV infects peripheral nerve endings from where it travels to the central nervous system (CNS) and eventually infects the brain.^7^^,^^8^ Once established in the CNS, massive viral replication will occur, inducing the onset of clinical symptoms and the inevitably fatal viral encephalitis. The clinical presentation varies, with characteristic but at times alternating symptoms of furious and paralytic rabies, complicating clinical recognition; particularly in cases lacking a clear exposure history.^9^^,^^10^ Incubation time from exposure to the onset of clinical, neurological, symptoms vary from five days to six months or longer, and RABV detection in this pre-clinical phase cannot be diagnosed as the virus remains undetectable. While antemortem diagnosis using biological specimens such as saliva, neck skin biopsies, and cerebrospinal fluid is possible in the clinical phase,^11^^,^^12^ diagnostic services are often unavailable in resource-poor settings, forcing reliance on presumptive clinical diagnoses.^5^^,^^13^

Progress towards global rabies elimination, driven by the WHO's “Zero by 30” initiative, is hindered by barriers to both human and animal vaccination, fragile health infrastructure, and logistical limitations in rabies surveillance.^14^ These challenges have been further exacerbated by global health crises such as the COVID-19 pandemic.^15^^,^^16^ Current rabies surveillance whether passive (based on patient or veterinary records), or active (involving animal testing), often falls short of providing the fine-scale epidemiological data needed to guide effective control strategies and may underestimate the true urgency of the situation.^5^^,^^17^^,^^18^ Conventional animal-based methods, including capturing and testing free-roaming dogs, are logistically demanding, costly, and often unreliable.^17^

Given this context, the development of innovative, cost-effective, and reliable surveillance tools is critical. Wound swabbing is a promising approach as it enables PCR-based detection of viral RNA from recent bite sites in a minimally invasive manner. In this prospective observational cohort study, we evaluate the potential of wound swab sampling from recent deep, unwashed dog-inflicted wounds in rabies-prone exposures in Karachi, Pakistan. The primary aim of the study was to detect RABV by PCR in wound swabs. Additionally, we characterised viral genetic diversity through sequencing and assessed levels of RABV neutralising antibodies in patients following intradermal PEP. Our findings provide preliminary data that can support future surveillance strategies in regions with a high burden of this preventable disease.

## Methods

### Study design and participants

One hundred patients seeking treatment for dog bites at the Rabies Prevention Center at Indus Hospital & Health Network, Karachi were included in a prospective observational cohort between 02.02.2023 and 18.11.2024. The study included individuals presenting with dog bite injuries of all ages that presented with fresh (less than 24 hours) WHO-defined Category 3 wounds (i.e., bleeding, transdermal, or multiple), that were unwashed and untreated with herbs or chemicals.

All patients were systematically screened and were excluded from the study when the patient participated in another clinical trial investigating a vaccine or drug in the 2 weeks preceding the study, planned participation in another clinical trial during the present study period, or received a vaccine in the 2 weeks preceding the study, with the exception for influenza vaccination and tetanus immunisation.

### Procedures

**Fig. 1:**
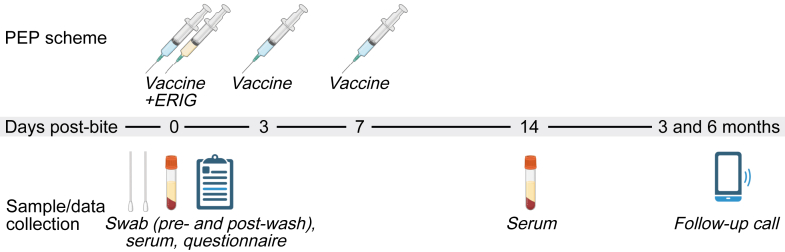
**Study outline.** PEP administration and sample and data collection was performed on the indicated days post-bite.

After explaining the study procedures and obtaining consent from the patient, parent, or guardian, individual bite wounds were swabbed using nylon-flocked swabs (Sorfa, Zhejiang, China) (Fig. 1). Cleaning of wounds was performed for 15 min with soap and water, as per WHO guidelines. Initially, only pre-wash wound swabs were collected from all participants. During the study, observing an unexpectedly high proportion of PCR-positive swabs, we added post-wash wound swabs for a subset of participants to evaluate the impact of wound washing on viral RNA detection. For patients with multiple bite wounds, separate swabs were collected from each wound using individual swab sticks. These multiple swabs were considered as a single exposure per patient, as the primary objective was to determine whether RABV RNA could be detected in the individual rather than in each wound separately.

From 21 non-bite swabs were taken from muscle-deep bleeding skin wounds in the Indus Hospital Emergency Department to serve as RABV-negative controls. All swabs were kept in 1 mL virus transport medium for future processing. After swabbing, all individuals received the first two doses of the six-dose intradermal RABV vaccination (Rabivax-S, 100 μl in both arms, deltoid muscle, Institut Pasteur du Cambodge (IPC) regimen^18^), and infiltration of equine anti-rabies immunoglobulins (ERIG, Equirab; Bharat Serums and Vaccines Ltd., India) in the wound as much as anatomically possible and around the wound only, with the maximum dosage being based on the weight of the patient. Administration of RABV vaccination and ERIG was part of standard care.

A short questionnaire was used to collect details, including the date of occurrence, age, gender, prior rabies vaccination, geographical area where the bite occurred, anatomical bite site, and the biting dog (e.g., pet/unknown, escaped/died, number of persons bitten by the same dog).

To measure virus-neutralising antibodies, venous blood was drawn before the first intradermal vaccine injection. The patients were instructed to return for subsequent intradermal vaccine administration on the third- and seventh-day post-bite, and for additional serum collection on day 14 to measure virus neutralising antibody levels against RABV. Three and six months after first presenting at the Rabies Prevention Center, the health status of the dog-bite patients was checked through individual phone calls. Sera and swabs were maintained at −80 °C until analysis or shipment to Erasmus MC, the Netherlands, where the laboratory analysis was performed as described below.

### Laboratory procedures

#### Swab processing and RABV RT-PCR

Swab-containing tubes were thawn, after which 500 μl of sample was mixed with 750 μl MagNa Pure 96 external lysis buffer (Roche Diagnostics, Almere, the Netherlands). Total nucleic acids were isolated (MagNA Pure 96, Roche, using the MP96 DNA and Viral NA large volume kit and protocol), after which the RNA was used in subsequent RT-PCR using TaqMan Fast Virus 1-step Mastermix (Thermo Fischer Scientific, Waltham, MA, USA) and RABV genotype-1 specific probes (110 bp product)^19^ as well as broadly reacting pan-lyssavirus probes (165 bp product).^20^ For both the genotype-1 and pan-lyssavirus RT-PCR the following PCR program was used: 5 min 50 °C, 20 sec 95 °C, followed by 45 cycli (3 sec 95 °C, 30 sec 60 °C).

#### Heminested RT-PCR and sequencing PCR

Swabs that showed a positive Ct (<45) on at least one of the RT-PCRs were confirmed in a subsequent sequencing PCR. A heminested broadly reactive lyssavirus PCR was used targeting the beginning of the N-gene (nucleotide position 55–641),^21^ using the OneStep PrimeScript III RT-PCR kit (Takara Bio Inc., Shiga, Japan) and using in-house optimised cycles for the first PCR (30 min 50 °C, followed by 40 cycles (2 min 94 °C/30 sec 94 °C, 30 sec 50 °C, 30 sec 72 °C, 605 bp product), 5 min 72 °C) and Q5 HiFidelity DNA Polymerase (Sigma Aldrich, St.Louis, MO, USA) for the second, nested, PCR (40 cycles (30 sec 98 °C/10 sec 98 °C, 30 sec 50 °C, 30 sec 72 °C)/2 min 72 °C, 581 bp product).

A sequencing PCR (25 cycles of 1 min 96 °C/10 sec 96 °C, 5 sec 50 °C, 1 min 60 °C) was performed on 1–40 ng of PCR product using the primers from the second, nested, PCR, and the BigDye terminator V3.1 Cycle Sequencing Kit (Thermo Fisher Scientific, Waltham, MA, USA). Products were purified using a Performa DTR V3 96-well plate (Edge Biosystems, Gaithersburg, MD, USA), after which sequencing was performed on an ABI/Hitachi 3500 genetic analyser (Applied Biosystems, Thermo Fischer Scientific, Waltham, MA, USA) using POP7. Initial sequence analysis was performed using SeqMan Pro software, version 16 (DNASTAR, Madison, WI, USA) to create.fas files of 527 nucleotides for phylogenetic analysis.

#### Phylogenetic analysis

All available RABV sequences present on the GenBank database on 28-07-2025 were downloaded, combined with our newly generated partial N-gene sequences and aligned using mafft. All unverified, patient sequences and partial genome sequences outside the N-gene were removed resulting in a total of 16,765 sequences which were included in the analysis. Phylogenetic analysis on the alignment of the 527 nucleotide region was performed using IQTREE2 (https://academic.oup.com/mbe/article/37/5/1530/5721363) using the GTR + F + R7 model as best predicted model. The resulting phylogenetic tree was visualised in FigTree v1.4.4 (https://tree.bio.ed.ac.uk/software/figtree/).

#### Rabies fluorescent antibody virus neutralisation test (rFAVN)

Serum samples were heat-inactivated for 30 min at 56° for prior to use in the rabies fluorescent antibody virus neutralisation test (rFAVN) and processed as described in the WHO laboratory guidelines.^22^ Sera were analysed in quadruplicate and microtiter plates were read blinded. The lower limit of detection for the rFAVN is 0.06 IU/mL, the upper limit of quantification is 13.77 IU/mL. Following WHO recommendations, the cut-off for protection was defined as 0.5 IU/mL.

### Statistical analysis

We used the Wilcoxon signed-rank test to determine differences in Ct values. To quantify the magnitude of these paired differences, we calculated Hodges-Lehmann median differences along with their corresponding 95% confidence intervals. The Wilcoxon-signed rank was chosen given the non-normality of the PCR data, as was initially assessed by the Shapiro–Wilk test. We calculated the proportion of individuals that had virus neutralising titres exceeding 0.5 IU/mL at day 0 and day 14. The 95% confidence interval of these proportions was based on the Wilson score interval.

### Sample-size estimation

This study was designed as a pilot feasibility study to evaluate rabies virus RNA detection in wound swabs, an approach that has not previously been described in humans. At the time of study design, no prior data were available on the expected sensitivity or detection rate of RABV RNA in human bite wounds in Pakistan, and no reliable surveillance data existed to inform a formal power calculation. Therefore, a pragmatic sample size of n = 100 was chosen based on feasibility considerations, patient flow at the Rabies Prevention Center, and the exploratory objectives of the study. Given the stringent inclusion criteria (WHO category III wounds, unwashed, and sampled within 24 h of exposure), the number of eligible patients was expected to be substantially lower than the total number of individuals presenting to the clinic with a dog-bite.

### Ethics statement

The study protocol (Appendix A) was approved by the Indus Hospital Institutional Review Board (IHHN_IRB_2022_11_014) and conducted in accordance with the ethical principles of the Declaration of Helsinki. Written informed consent was obtained from all participants or their parents/guardians and included the publication of the clinical image presented in Fig. 3A.

### Role of the funding source

The funders of the study had no role in study design, data collection, analysis, or interpretation, or writing of the report.

## Results

### Participant demographics and bite characteristics

**Fig. 2:**
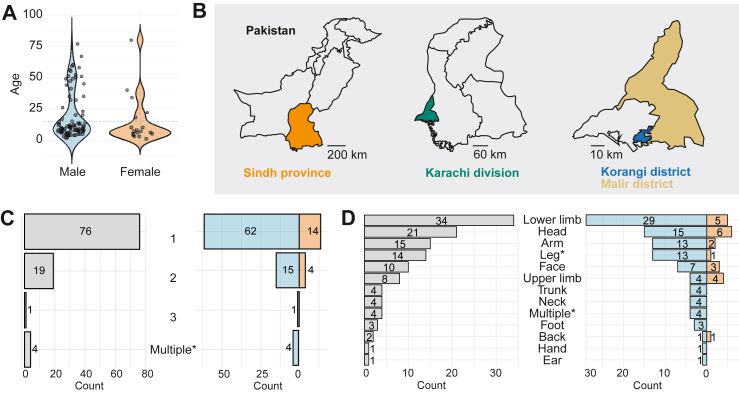
**Age, geographical distribution, and wound characteristics of study participants.** A) Age and gender distribution of the included participants. B) Geographical distribution throughout the different districts and subdivisions of Karachi. C) Number of anatomical bites sites of all included patients (left) or split per gender (right, male in blue and female in orange). D) Anatomical bite sites of all included patients (left) or split per gender (right, male in blue and female in orange).

100 individuals presenting with dog-bite injuries were included in the study between 02.02.2023 and 18.11.2024, of which 82 were males (mean age 19.8, range 1–80, Table 1, Fig. 2A) and 18 females (mean age 14.9, range 1–77). In this period, a total of 18,870 dog-bite patients were treated at the clinic, of whom 5456 presented with category III wounds. Of these 5456 patients, the majority had already washed or treated their wounds prior to presentation. Only patients who met the inclusion criteria (unwashed WHO category III wounds and presentation within 24 h after exposure) were invited to participate; no additional selection criteria based on the dog's behaviour or perceived rabies risk were applied.

**Table 1:** Sex and age distribution of the study participants.

	N	Age				Under 15	
		Mean	Median	Min	Max	n	%
Male	82	19.8	11	2	77	54	65.9
Female	18	14.9	7	1	80	13	72.2
Total	100	18.9	10	1	80	67	67.0

67 of the 100 study participants (54 males and 13 females) (67%) were younger than 15. All study participants were residents of the Karachi division (Sindh province), with the majority originating from the Korangi (84/100, 84%) and Malir districts (10/100, 10%, Fig. 2B). Notably, the Indus Hospital—where this study was conducted—is in the Korangi district, which, along with the adjacent Malir district, contributed to the highest proportion of the patients. This is largely attributable to the hospital's location and the strict inclusion criteria (within 24 h after the initial bite accident), which were most met by individuals from these two districts due to their proximity to the hospital.

Most study participants (n = 76) presented with a single anatomical bite site (Fig. 2C), while a smaller number had two (n = 19), three (n = 1), or multiple (unspecified, n = 4) bite sites. Bites to the lower limbs were most common (n = 34; Fig. 2D), followed by bites to the head (n = 21) and the arms (n = 10). The distribution of the number of bite sites and their anatomical locations was similar between male and female participants, and no clear differences were observed between younger and older individuals.

### Detection and phylogenetic analysis of RABV RNA in bite wounds

**Fig. 3:**
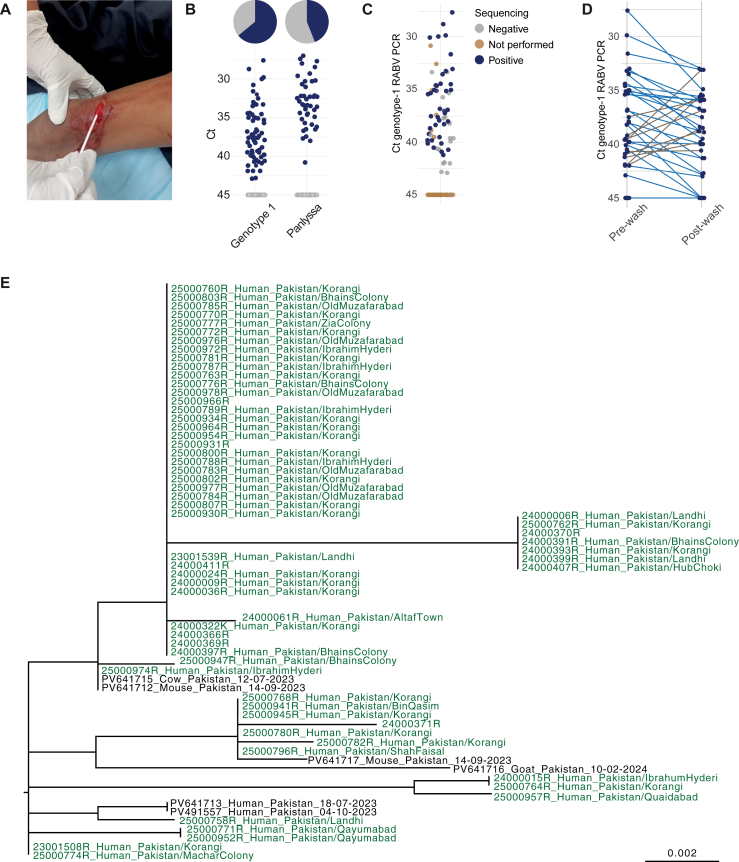
**Molecular analysis of wound swabs.** A) Representative example of a dog-bite wound and the swabbing procedure. B) Ct values of the Genotype-1 RABV PCR (left) and the Pan-lyssa RABV PCR (right) of the RNA extracted from the dog-bite wound swabs. Dots represent individual sera, colors indicate a positive Ct (<45, blue), or a negative Ct (45, grey). C) Visualization of the confirmation of RABV RNA through sequencing. D) Comparison of the Ct of the Genotype-1 PCR in the pre-wash dog-bite wound swabs and the post-wash dog-bite wound swabs. Paired samples of individual patients connect with a line, that indicates an increase in Ct (blue) or a decrease in Ct (brown). E) Phylogenetic analysis of the RABV N-gene sequences obtained from the wound swabs (green). Reference sequences are indicated in black. All sequences cluster with the RABV sublineage Arctic-like 1a.

Swabs were taken from individual wounds (Fig. 3A) and processed for RABV RNA detection by RT-PCR. In patients with multiple wounds, swabs were taken from each wound, using separate swab sticks but these were collected in a single tube of VTM and counted as a single exposure. In 64 out of 100 individuals that were tested before wound washing RABV RNA was detected using the Genotype-1 RABV PCR (Fig. 3B), with a mean Ct value of 36.9 and values ranging from 27.6 to 43. Using the pan-lyssa PCR, RABV RNA was detected in 44 out of 100 individuals, with a mean Ct of 33.1 and values ranging from 27.0 to 40.8. All swabs that tested positive in the Genotype-1 RABV PCR tested positive as well in the pan-lyssa PCR (Supplementary Figure S2, Appendix B). The Ct values of samples estimated using the Genotype-1 RABV PCR were slightly higher than the corresponding values of the pan-lyssa PCR (median difference 0.2, 95% CI –1.0 to 1.5). RABV RNA was not detected in any of the control swabs from non-bite wounds (Supplementary Figure S1, Appendix B).

Of the 64 swabs in which RABV genome was detected by either PCR, 40 could be sequenced (Fig. 3C). Ct levels of sequence-confirmed PCRs ranged between 27.6 and 41.2, with 25 samples showing a Ct above 35. To assess the effect of wound washing, swabs were collected from a subset of participants both before and after washing and analysed for RABV RNA by PCR. Among 55 dog-bite participants with paired swabs, RABV RNA was detectable in the pre-wash swabs of 33 individuals (Ct < 45). Of these 33 paired samples, 24 post-wash swabs showed an increase in Ct, suggesting a reduction in viral RNA at the wound site (Fig. 3D).

Overall, wound washing was found to increase the Ct values of the swabs by 1.5 Ct values (95% CI 0.2–2.7). In three swab pairs, the amount of RABV RNA was undetectable after wound washing. Nine swab pairs showed a decrease in Ct, suggesting an increase in viral load. However, all these samples had a high Ct in the pre-wash swab (mean Ct of 37, range 33.1–40.6) indicative of a low viral load, and therefore the results should be interpreted with caution.

Phylogenetic analysis of the retrieved viruses showed that all sequences clustered with recent (2023–2024) isolates from Pakistan (Fig. 3E). For broader regional context, sequences from neighbouring countries were included in the Supplementary Figure S3, Appendix B. Only minor (up to 11 single-nucleotide polymorphisms SNPs, corresponding to 97.9% similarity) variation was observed between the sequences obtained from the swabs.

### Evaluation of neutralising antibodies and PEP response

**Fig. 4:**
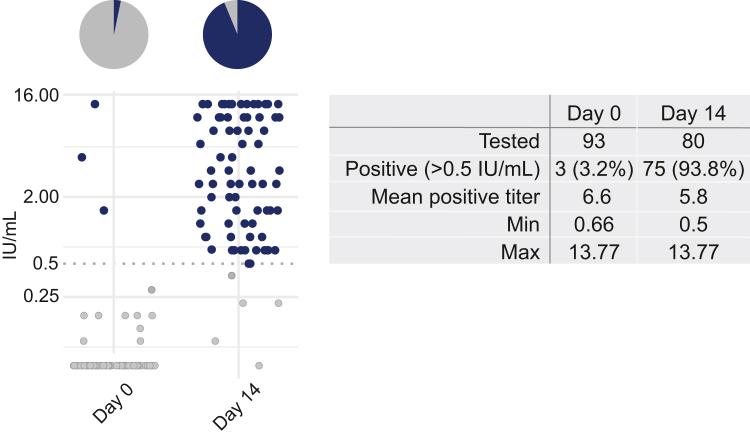
**Serological analysis of sera from PEP-recipients**. Virus-neutralization tests (VNTs) were performed before receiving PEP (Day 0) and fourteen days later (Day 14). The threshold for a protective titer (0.5 IU/mL) is indicated by a dotted line, and the graph shows individual positive (blue) and negative (grey) sera. Neutralizing titers are plotted on a log2-scale for clarity.

Serum was collected from individuals presenting with dog-bite injuries before receiving the first vaccination (t = 0) and fourteen days later (t = 14), corresponding to seven days after receiving the third and final dose of PEP. Serum samples were available from 93 to 80 participants at t = 0 and t = 14, respectively; 13 patients were lost to follow-up. A positive virus-neutralising antibody (VNA) titre (>0.5 IU/mL) was detected at t = 0 in three participants (3.2%, 95% CI 1.1–9.1%) (Fig. 4). At t = 14, neutralising antibody titres above the WHO protective cut-off of 0.5 IU/mL were detected in 72 of 77 participants (93.8%, 95% CI 86.2–97.3%) who were seronegative at baseline. Overall, 75 of 80 tested samples (93.8%) had VNA titres >0.5 IU/mL at day 14. The mean positive titre was 5.8 IU/mL, ranging from 0.5 to >13.77 IU/mL. The five participants whose VNA titres remained below 0.5 IU/mL at day 14 were all from out-of-town locations. Despite repeated attempts, we were unable to convince them to return for follow-up testing, and no additional samples could be collected to assess subsequent antibody development.

Follow-up of study participants was performed by individual phone calls; successful contact was achieved in 54% of the individuals due to incorrect phone numbers or lack of response. Among those contacted, all (100%) reported that they were doing well and none had developed signs suggestive of rabies.

## Discussion

Despite the global recognition of rabies as a neglected disease, the true scale of its impact remains elusive due to substantial gaps in diagnosis and documentation.^5^^,^^13^ This challenge extends to accurately quantifying virus circulation within animal reservoirs and human populations, hindering effective surveillance.^17^^,^^23^^,^^24^ Reliable and timely epidemiological data are indispensable for guiding policy decisions and allocating resources efficiently. Therefore, there is an urgent need to develop and implement innovative surveillance methodologies capable of overcoming the limitations of current approaches. In the presented study we show the detection of RABV RNA in wound swabs collected from individuals presenting with dog-bite injuries in the rabies-endemic region Karachi, Pakistan. Data on human rabies cases in Pakistan is limited,^25^^,^^26^ and while recent numbers are lacking, reports have shown the decrease of reported human cases from 2549 in 1990 to 928 in 2016,^27^ a number that seemed to stabilise in 2019 with an estimate of 1198 human cases.^28^ This decrease can be attributed to raising awareness, well-organised and specialised dog-bite clinic in combination with the availability of PEP, and the general shift from using sheep-brain derived vaccines to tissue-culture vaccines.^29^, ^30^, ^31^

Besides the incomplete data on rabies in humans, insights on RABV circulation in dog populations are missing as well. Despite attempts to eliminate the disease in roaming street dogs through massive culling or private vaccination efforts,^32^ large and continuous control measurements are not in place.

Currently, most conventional rabies diagnostic and surveillance methods are designed to detect clinical disease, rather than early exposure. Our study demonstrates that RABV RNA can be detected directly from bite wounds, highlighting a complementary approach that may identify recent exposures before the onset of symptoms. While this method does not replace standard diagnostic testing, it provides valuable insight into viral circulation and potential risk at the point of exposure.

We observed a high proportion of RABV RNA–positive wound swabs in our study cohort, which may reflect substantial local virus exposure. However, this finding should be interpreted cautiously: our cohort included only fresh, unwashed category III bite wounds, and does not represent the general population of individuals presenting dog-bite injuries. Due to limited surveillance data in Pakistan, direct comparisons with other studies are not possible. Importantly, while thorough WHO-guideline wound washing reduced viral RNA levels, virus remained detectable in most wounds, reinforcing the critical role of the dog-bite and PEP clinics while simultaneously serving as a warning signal to initiate dog vaccination campaigns in the area. While we show the presence of RABV RNA in a high number of individuals with category III dog-bites this does not necessarily mean that these individuals will develop rabies. Disease development depends on multiple factors, including viral load, anatomical location of exposure, proximity to nerve endings, and the host immune response.

Consistent with previous reports, we observed that most individuals with dog-bite injuries in our study were males (82%), with a substantial proportion under 15 years of age. This aligns with documented patterns of rabies exposure, where middle-aged males and children are often at higher risk due to increased outdoor activity and contact with free-roaming dogs.^26^^,^^33^, ^34^, ^35^ These demographic trends underscore the need for targeted public health interventions, including education on bite prevention and timely post-exposure prophylaxis in high-risk groups.

PCR-based analysis of bite-wound swabs is an elegant and minimally invasive approach for acquiring essential rabies surveillance data. Given the large number of individuals that generally present at dog-bite clinics each day, detailed and longitudinal insights on virus circulation can be generated. While molecular detection of pathogens from wounds is well-established for various bacterial infections^36^^,^^37^ and virus-specific lesions like herpes simplex^38^ it is not a common practice for bite wounds.

A limitation of our study is the lack of diagnostic confirmation of the biting animals. Without systematic testing or observation, it is not possible to determine which wounds were inflicted by truly rabid dogs, and therefore the RT-PCR positivity rate in wound swabs cannot be directly extrapolated to estimate the true rabies burden in the community. Linking human wound swab results to animal testing would strengthen the epidemiological interpretation of these findings; however, systematic animal surveillance in this setting is challenging due to logistical, resource, and safety constraints. Despite these limitations, the detection of RABV RNA in a substantial proportion of unwashed bite wounds demonstrates the feasibility of this approach for human exposure monitoring. Future research should explore integrated human–animal surveillance strategies, potentially combining wound swab testing with targeted observation or sampling of suspect animals, to better inform rabies surveillance and control programs in endemic regions.

In our study, the pan-lyssavirus PCR showed a lower positivity rate compared to the genotype-1–specific assay. The pan-lyssavirus PCR is optimised for broad detection across lyssavirus species and may fail to detect low-titre RABV RNA in some wound swabs. To confirm that high Ct values corresponded to true presence of viral genome, sequencing was performed on all PCR-positive swabs using a sequencing PCR. Even for Ct values approaching 40, indicating very low amounts of viral RNA, genomic information could be successfully generated. This is particularly important for identifying circulating lineages and assessing genomic diversity within ongoing outbreaks.

Phylogenetic analysis revealed only minor variation between sequences, with no clear clustering by geographic location or sampling time. Due to low levels of detected viral genome in the wound swabs, amplicon-based sequencing of a single PCR product was performed rather than whole-genome sequencing. Of 64 PCR-positive swabs, 40 could be successfully sequenced. As RNA integrity was confirmed to be high in all samples, the lower sequencing success is attributed to limited viral RNA levels in these swabs, which reduced the efficiency of nested PCR amplification and downstream sequencing.

Although the nucleoprotein is not the most variable gene of RABV and some variation may therefore have gone undetected, it is a well-established target that is widely used for strain and lineage determination due to its reliable detection.^39^ All sequenced viruses belonged to the Arctic-like 1a sub lineage, the predominant RABV lineage reported in Pakistan, confirming that the detected viruses are representative of locally circulating strains. No clear clustering by geography or sampling time was observed; however, as most samples originated from the Korangi and Malir districts of Karachi, these findings should be interpreted cautiously. The limited spatial and temporal diversity of the dataset may preclude meaningful conclusions about broader geographic or temporal patterns.

Wound washing is the first and an essential step in post-exposure rabies prevention, as recommended by the WHO. In our study, comparison of pre- and post-wash swabs showed that washing reduced the levels of detected viral RNA in most wounds, with the greatest reductions observed in wounds that initially had higher detectable RNA levels (presumably those at highest risk of infection). However, in general the differences were relatively small, it is important to note that our assay is not quantitative, and results can be influenced by how thoroughly the swab is collected. Importantly, viral RNA was still detectable in many post-wash swabs, reinforcing that wound washing alone is insufficient to prevent rabies, and timely post-exposure prophylaxis (PEP) remains crucial.

PCR on wound swabs is intended as a research and surveillance tool, not a diagnostic test. Its reliability is affected by timing, sample quality, and assay sensitivity, meaning that a negative result cannot rule out infection. Therefore, wound washing and PEP should never be delayed to allow for swab collection. Future studies should further validate the use of wound swab PCR in epidemiological monitoring and to explore its potential role in understanding rabies exposure in endemic settings.

Virus neutralising antibodies were detected in 72 out of 77 study participants (93.5%) that had a negative (<0.5 IU/mL) titre at day 0, indicating the success of the three-dose intradermal PEP regime, as the WHO advice.^40^^,^^41^ The detection of neutralising antibodies on day 14 could result from vaccination, administration of rabies immunoglobulins (RIG), or a combination of both, and this cannot be distinguished with certainty. Although RIG was infiltrated locally into the wounds primarily to neutralise virus at the site of exposure, some systemic absorption cannot be completely excluded and may contribute to measurable VNA titres. Therefore, we cannot definitively conclude that the observed antibodies represent true vaccine-induced seroconversion.

Standard practice for assessing PEP adequacy is to measure VNA titres 14 days after the first dose.^42^ However, this time point may be relatively early, and it is expected that most individuals would develop protective titres if tested later.^43^ Low-level positive VNA titres observed in three participants prior to vaccination are likely due to either unreported prior vaccination or past subclinical RABV exposure. Although rare, the presence of low-level positive VNA titres in unvaccinated individuals has been documented in several studies, potentially reflecting subclinical exposures.^44^, ^45^, ^46^

In conclusion, our study highlights wound swabbing as a promising and practical approach for rabies surveillance in endemic settings. RABV RNA was detected in a high proportion of fresh, unwashed category III bite wounds, demonstrating that viral genetic material can be recovered directly from human exposure sites using PCR-based methods. However, this proportion cannot be extrapolated to the general dog-bite population, as our stringent inclusion criteria deliberately enriched the study cohort for recent, untreated, high-risk exposures. In the absence of systematic information on the infection status of the biting dogs, no conclusions can be drawn regarding diagnostic sensitivity, test performance, or true population prevalence. Nevertheless, PCR-based wound swabbing circumvents the need for high-containment laboratories and specialised expertise required for conventional animal-based rabies diagnostics and may therefore represent a valuable complementary tool for monitoring RABV circulation in settings where routine animal surveillance is limited or absent. The minimally invasive nature of swab collection and the ability to generate viral sequence data further support its potential utility for strengthening molecular surveillance and improving understanding of rabies virus circulation in endemic regions.

## Contributors

C.W.E.E., N.S., M.A.G., J.A., and C.H.G. designed the study and developed the study protocol. Patient recruitment and clinical study procedures in Pakistan were conducted by M.A.G. and N.S., with logistical and laboratory support from F.N. and S.H. J.A. coordinated laboratory activities at Indus Hospital. At Erasmus MC, K.A. supervised neutralisation assays, while J.J.C.V., J.G., and B.B.O.M. conducted molecular testing, sequencing, and data analyses. Data interpretation was performed by C.W.E.E., N.S., M.A.G., B.B.O.M., J.A., and C.H.G. C.W.E.E., C.H.G., B.B.O.M., and J.J.C.V. verified the underlying data. The manuscript was drafted by C.W.E.E., N.S., M.A.G., J.A., and C.H.G., and all authors reviewed and approved the final manuscript.

## Data sharing statement

Individual participant data that underlie the results reported in this article, after de-identification, will be made available immediately following publication to anyone who wishes to access the data. Proposals should be directed to c.embregts@erasmusmc.nl to gain access.

## Editor note

Map lines delineate study areas and do not necessarily depict accepted national boundaries.

## Declaration of interests

The authors declare no competing interests.

## Appendix A Supplementary data

Ethical Approval

Supplementary Figure S1 Ct values of the Genotype-1 RABV PCR (left) and the Pan-lyssa RABV PCR (right) of the RNA extracted from the dog-bite wound swabs. The lines connect the paired specimen.

Supplementary Figure S2 Ct values of the Genotype-1 RABV PCR (left) and the Pan-lyssa RABV PCR (right) of the RNA extracted from the control wounds. Negative Ct (45) is depicted as grey, the pie charts demonstrate 100% negative results.

Supplementary Figure S3 Phylogenetic analysis of the RABV N-gene sequences obtained from the wound swabs (green). Reference sequences are indicated in black.

Final Research Proposal
