# Supplementary material for: Detection of rabies virus RNA in dog-bite wounds in a rabies-endemic area: evidence from an observational cohort study
Source: eBioMedicine. 2026 Apr 10;127:106250. doi: 10.1016/j.ebiom.2026.106250 (PMC13174237; doi:10.1016/j.ebiom.2026.106250)
Supplement: Ethical Approval [file mmc1.pdf]

Indus Hospital & Health Network
Institutional Review Board
IHHN IRB#1 Biomedical/Clinical
IHHN IRB#2 Social/Behavioral

Indus Hospital Research Center
Indus Hospital & Health Network,
Korangi Crossing
Karachi 75190, Pakistan
www.indushospital.org.pk

Protection of Human Subjects – Declaration / Assurance of IRB Approval

PI	IHHN-IRB #	Department/Institute
Dr. Naseem Salahuddin	IHHN_IRB_2022_11_014	Infectious disease/IHHN
**Approval Date**	**Expiration Date**	**Administrative Due Date**
17-Jan-23	16-Jan-24	17-Dec-23
**The following research study has been reviewed by the IHHN-IRB:** Serum sampling of dog-bite victims in Karachi, Pakistan, to investigate the pre- and post- RABV PEP treatment immune response		

IRB EXPEDITED STATUS: APPROVED

The IHHN-IRB has reviewed the above-referenced study and determined that, as currently described, it was eligible for expedited review and has been approved, as per the following category:

Category(2): Collection of blood samples by finger stick, heel stick, ear stick, or venipuncture

Stamped consent form(s) [if applicable] are attached for your reference.

As principal investigator for a study involving human subjects, you assume certain responsibilities, specifically:

1. You will conduct the study according to the protocol approved by the IRB. As the PI, you will be accountable for your own research and the protection of human subjects. You will ensure, at all times, that you have the appropriate resources and facilities to conduct the study. You will ensure that all research personnel involved in the conduct of the study have been appropriately trained on the protection of human subjects, in addition to the study procedures.

2. Any unanticipated problems involving risks to participants or others will be reported to the IRB in accordance to the IRB policy. Changes in approved research initiated without IRB approval to eliminate apparent immediate hazards to the participant, are to be reported to the IRB.

3. Any changes in your research plan must be submitted to the IRB for review and approval prior to implementation of the change. Proposed changes in approved research cannot be initiated without IRB approval, except when necessary to eliminate apparent immediate hazards to participants.

This letter does not require to be signed by IRB Chair or designee.

Office of Research, Innovation and Commercialization (ORIC)
Indus Hospital & Health Network,
Korangi Crossing
Karachi 75190, Pakistan
www.indushospital.org.pk

Protection of Human Subjects – Modification Approval no 01

PI: Dr. Naseem Salahuddin
Dept/Institute: Infectious disease/IHHN

From: Chairperson IRB

**Approval Date**:	**Expiration Date:**	**Administrative Due Date:**
28-Nov-24	28-Nov-25	28-Oct-25

Research Study Title: Serum sampling of dog-bite victims in Karachi, Pakistan, to investigate the pre- and post- RABV PEP treatment immune response
IHHN-IRB Number: IHHN_IRB_2022_11_014

MODIFICATION: APPROVED

The IHHN-IRB has reviewed and approved the modifications requested for the above-referenced human-subjects research project on 28-Nov-24.

The modification approval is for the following revisions to the study:

Change in consent form: 'informing patients that their blood and swab samples may be sent to Erasmus University in the Netherlands.' Within the consent form.

It is inclusive of the following documentation:

- All previously approved documents

As a reminder, no other changes to the study may be implemented without prior IHHN-IRB review and approval. This approval is limited to the activities described in the study protocol and the approved modifications. The action taken on this study does not change the IRB expiration date listed above.

If you have any questions regarding this approval, please contact IHHN-IRB via email irb@tih.org.pk.

*** This letter does not require to be signed by IRB Chair or Designee. ***
