# Supplementary figures and images for: Detection of rabies virus RNA in dog-bite wounds in a rabies-endemic area: evidence from an observational cohort study
Source: eBioMedicine. 2026 Apr 10;127:106250. doi: 10.1016/j.ebiom.2026.106250 (PMC13174237; doi:10.1016/j.ebiom.2026.106250)

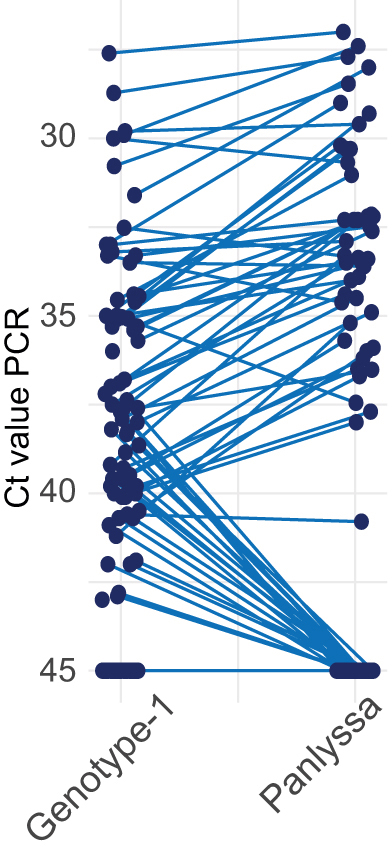

Supplement: Supplementary Figure S1 — Ct values of the Genotype-1 RABV PCR (left) and the Pan-lyssa RABV PCR (right) of the RNA extracted from the dog-bite wound swabs. The lines connect the paired specimen. [file figs1.jpg]

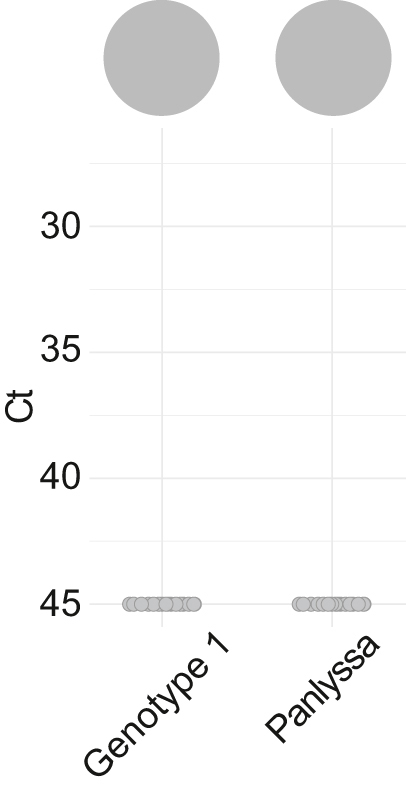

Supplement: Supplementary Figure S2 — Ct values of the Genotype-1 RABV PCR (left) and the Pan-lyssa RABV PCR (right) of the RNA extracted from the control wounds. Negative Ct (45) is depicted as grey, the pie charts demonstrate 100% negative results. [file figs2.jpg]

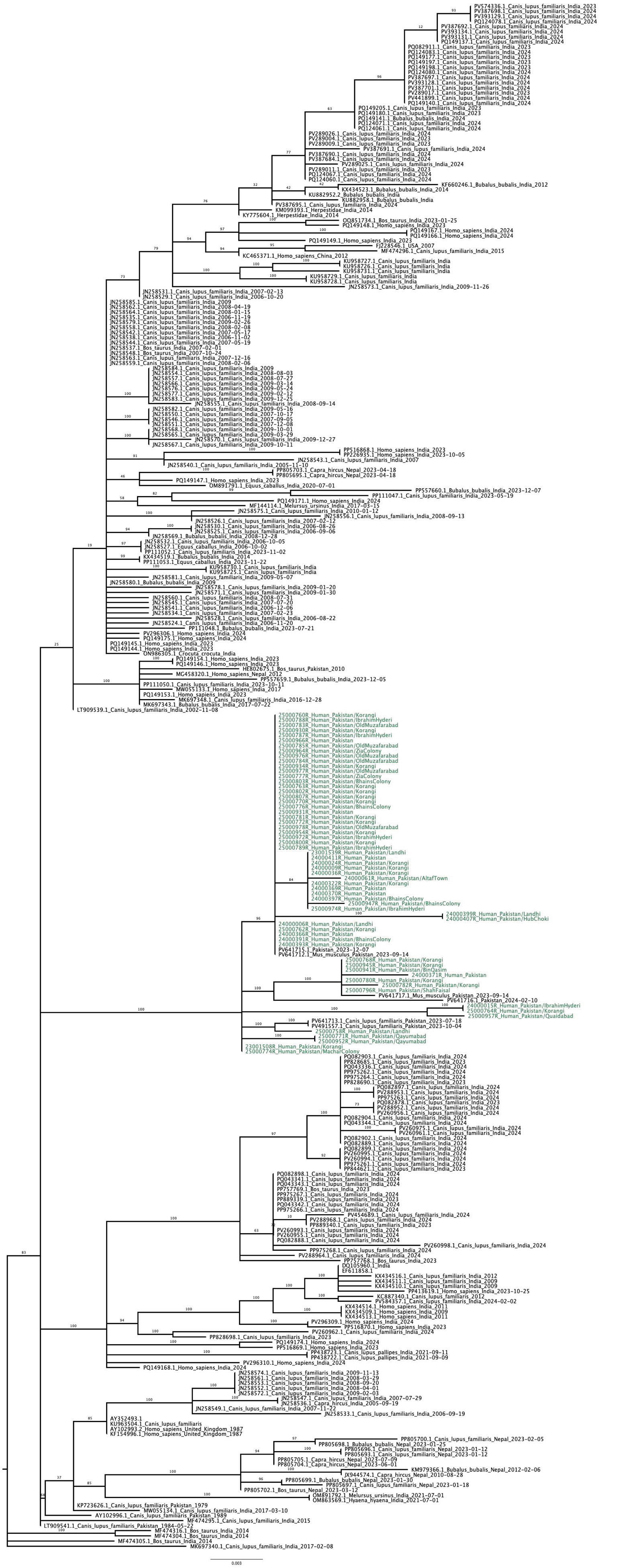

Supplement: Supplementary Figure S3 — Phylogenetic analysis of the RABV N-gene sequences obtained from the wound swabs (green). Reference sequences are indicated in black. [file figs3.jpg]
