# Supplementary material for: Detection of rabies virus RNA in dog-bite wounds in a rabies-endemic area: evidence from an observational cohort study
Source: eBioMedicine. 2026 Apr 10;127:106250. doi: 10.1016/j.ebiom.2026.106250 (PMC13174237; doi:10.1016/j.ebiom.2026.106250)
Supplement: Final Research Proposal [file mmc2.pdf]

Serum sampling of dog-bite victims in Karachi, Pakistan, to investigate the pre- and post-RABV PEP treatment immune response

Introduction

Rabies virus is a deadly zoonotic virus belongs to the Rhabdoviridae family and the *Lyssavirus* genus, with a broad geographical range, causing outbreaks all over the world (1). Infection results in a viral encephalitis that is 100% fatal in humans as well as in most of the infected animals (2). Globally approximately 59,000 people worldwide die of rabies mostly in the developing countries of Asia and Africa., and 50% of these deaths were children under 15 years of age (3). In these countries the prevention and control of rabies are hindered because of inadequate medical treatment, insufficient vaccines and immunoglobulins, and the lack of attention from the public, resulting in a large number of reported cases of rabies (4, 5). Clinical symptoms only arise when the virus has reached the brain, after which no treatment options are available. A RABV-specific immune response is rarely detected during infection, and the virus is able to actively evade and suppress the hosts immune response. While most RABV research has focussed on the brain, knowledge on the first phases of the infection and the relation immune reaction against the virus is largely lacking. This knowledge is of great importance, given that the virus meets innate immune cells in the early phase of infection.

Rationale:

In this study we aim to characterize the early systemic cytokine response in dog-bite victims in Karachi, Pakistan, in order to investigate if and how the immune system is suppressed very early after RABV exposure. A first serum will be taken prior to PEP treatment, to investigative if there is an early cytokine response that would indicate immune activation or immune suppression. A second serum will be taken during the last follow up visit for PEP treatment, to analyse the strength of the immune reaction (neutralizing antibodies and cytokines)(5) against the PEP treatment. Additionally, given that early diagnostics are lacking in rabies, we will investigate if a swab from the bite wound area early after infection is a reliable way of confirming exposure to RABV.

Objectives:

The key objectives of this research are:

1. To characterize the early immune response against RABV in the serum of dog-bite victims

2. To quantify the immune response after receiving RABV PEP treatment
3. To investigate if RABV genome can be detected in a swab from a dog bite wound

Study population: The study population consists of dog bite victims that are visiting the Karachi Hospital clinic for RABV PEP treatment after a bite from a rabies-suspected dog.

Intervention: A serum sample and a swab from the wounded area will be collected before wound scrubbing and PEP treatment. A second serum sample will be collected when the patients visit the clinic to receive the final dose of PEP.

Main study parameters:

- Serum cytokine response (e.g. interferons, inflammatory response)
- Detection of RABV by PCR of swabs taken from the bite wound
- Antibody titers and cytokine response after receiving RABV PEP treatment

Laboratory techniques:

- Flow cytometry-based 13-plex bead assay (Biolegend) to quantify serum cytokines levels
- Pan-lyssa PCR to detect virus in wound swabs
- ELISA for rabies antibodies
- Virus neutralization assays (VNT) to detect neutralizing antibody levels in serum

Study design

Samples (serum, wound swab) will be taken from dog bite victims visiting the clinic for wound scrubbing and PEP treatment. To be included in the study the individual should present with a recent (<24h) type-3 exposure, without a washed or treated wound area. Swab sample: (1)To be taken once as soon as possible after bite and (2) Multiple swab samples will be taken if there are multiple category 3 wounds and (3) It will be taken from the dog bite wound before wound scrubbing and cleaning. A short questionnaire will be used to collect details about the patients (e.g. age, gender) and the bite (e.g. location, severity, day/time of bite).

Swab sample will be stored in the RNA-stabilizing buffer RNA later for later processing for PCR to determine the presence or absence of viral RNA. Samples will be stored in -80 degrees until analysis or shipment to the Erasmus MC, the Netherlands.

A serum sample will be taken before receiving PEP, as well as before receiving the last round of PEP. Sera will be stored at -20 degrees until analysis or shipment to the Erasmus MC, the Netherlands. Serum cytokines will be quantified with 13-plex bead assays and RABV antibodies will be quantified by ELISA and virus-neutralization assays (VNTs) or relatable assays.

Differences in serum cytokines are expected to be subtle, with large patient-to-patient variations. Given this, we aim to include serum of 100 dog-bite victims in this study.

Inclusion criteria

1- Dog bite victims of either gender and all ages.

2- Dog bite vicitms with WHO defined Category 3 wounds.

3- Dog bite victims with "probable rabies exposure"(Probable rabies exposure is when bitten by a dog/animal shows abnormal behavior with excessive salivation/bit without a reason/ the animal has bitten multiple people or other animals around).

4- Presented to Rabies Prevention Center with an open wound and with in an appropriate time when swab sample from the wound can be taken

Exclusion criteria

1- Washed wound, cleaned with disinfectant or applied any remedies on wound site prior visitng Rabies Prevention Center.

2- Participation in another clinical trial investigating a vaccine, drug, medical device, or medical procedure in the 2 weeks preceding the trial

3- Planned participation in another clinical trial during the present trial period

4- Receipt of any vaccine in the 2 weeks preceding the trial, except for influenza vaccination and tetanus immunization (related only to current animal bite exposure)

Statistical Analysis

Statistical analyses will be performed using SPSS version 24.0. Quantitative variables like gender will be reported in frequency/percentage. For qualitiative variables like age, cytokine level, antibodies titer will be reported as mean (STD) or median (IQR) as appropriate on the basis of normality. Paired sample t test will be used to compare the cytokine and antibodies titer in pre and post PEP treatment cohort. P value ≤ 0.05 will be considered as significant.

Ethical Consideration

Data Privacy and Security:

We will use the medical record numbers of the patients initially to screen data for duplication and eligibility criteria. Once the data has been cleaned, it will be de-identified and only this de-identified data will be analyzed.

The data will be kept on the secure servers of The Indus Hospital on password protected files that would be accessible only to the IRB approved study personnel. The results will be published in a de-identified form and no personal information will be shared.

Benefits and risk

Benefit

This study will lead to 1) better understanding of the early immune suppressive mechanisms by RABV 2) quantitative insights into the humoral immune response after receiving PEP, and 3) insights on the reliability and feasibility of taking a wound swab as an indicator of RABV-exposure through a dog bite. Understanding how RABV suppresses the immune response, and the effectiveness of PEP treatments, is essential in optimizing current PEP strategies and developing novel post-exposure treatments. Furthermore, early diagnostics for rabies are lacking, and the use of a wound swab to determine the presence or absence of RABV viral RNA will be of great added value.

Risk:

There is always a risk of loss of confidentiality of data. We will take all possible measures to ensure that all patient information is secure by de-identifying the data to be analyzed, storing this de-identified data on password protected files on IHHN secure servers and allowing access only to IRB approved study team.

1. Callaway HM, Zyla D, Larrous F, de Melo GD, Hastie KM, Avalos RD, et al. Structure of the rabies virus glycoprotein trimer bound to a prefusion-specific neutralizing antibody. 2022;8(24):eabp9151.
2. Hampson K, Coudeville L, Lembo T, Sambo M, Kieffer A, Attlan M, et al. Estimating the global burden of endemic canine rabies. 2015;9(4):e0003709.
3. Organization WH. WHO expert consultation on rabies: third report: World Health Organization; 2018.
4. Stokstad E. Taming rabies. American Association for the Advancement of Science; 2017.
5. Wilde H, Ghai S, Hemachudha TJV. Rabies: Still a silent killer targeting the poor. 2017;35(18):2293-4.
